# Self-management of persons living with diabetes mellitus type 2: Experiences of diabetes nurse educators

**DOI:** 10.4102/hsag.v25i0.1381

**Published:** 2020-11-17

**Authors:** Coleen O’Brien, Dalena van Rooyen, Esmeralda Ricks

**Affiliations:** 1 Day Clinic/Gastroenterology Unit, Greenacres Hospital, Port Elizabeth, South Africa; 2 Faculty of Health Sciences, Nelson Mandela University, Port Elizabeth, South Africa

**Keywords:** diabetes nurse educators, experiences, diabetes mellitus type 2, patient education, self-management

## Abstract

**Background:**

The global pandemic of diabetes mellitus type 2 (DM2) is the direct cause of significant health and economic problems for both governments and individuals owing to the high level of morbidity and mortality. South Africa has the second highest incidence of DM2 in sub-Saharan Africa.

**Aim:**

This article describes the experiences of diabetes nurse educators in relation to self-management of DM2 of persons living with DM2.

**Setting:**

This article involves nurse educators working in diabetes clinics in public and private hospitals in Nelson Mandela Bay Municipality, South Africa.

**Methods:**

A semi-structured interview guide was used to collect data from a focus group interview comprising three participants and two individual interviews. The interviews were recorded with the permission of participants, transcribed and then coded using Tesch’s model of data analysis. Themes were agreed upon with the consensus of the researcher, two supervisors and an independent coder.

**Results:**

Three themes were identified: (1) diabetes nurse educators have clear perceptions about the importance of self-management of DM2 of persons living with DM2, (2) there are factors that affect persons living with DM2 with respect to self-management and (3) there are ways in which professional nurses can assist persons living with DM2 in the self-management of their condition.

**Conclusion:**

This article identified factors that diabetes nurse educators experienced in either assisting or hindering patients in self-management of their DM2. The diabetes nurse educators voiced their views on how professional nurses can provide support to the patients living with DM2.

## Introduction

The increasingly widespread pandemic of diabetes mellitus type 2 (DM2) is the direct cause of significant health and economic problems for both governments and individuals living with DM2. Diabetes mellitus type 2 is one of a group of metabolic disorders characterised by raised blood glucose levels, resulting in insulin deficiency or insulin resistance (Catsicas 2009). Persons living with DM2 are dealing with a chronic condition that has an extremely high rate of morbidity and mortality. To demonstrate the rapid increase of the condition, in 2015, it was estimated that 415 million people worldwide were living with DM2, a figure that had increased to an estimated 463 million by 2019. The projected global increase of people living with DM2 for 2040 was 640 million in 2015, but by 2019, the global estimate was expected to rise to 700 million (Saeedi et al. 2019; Unnikrishnan et al. 2017). The International Diabetes Federation (IDF) has estimated that in Africa the incidence will rise from 14.2 million in 2015 to 34.2 million in 2040 (Mutyambizi et al. 2018). South Africa has the second highest incidence of DM2 in sub-Saharan Africa, with an estimated prevalence of 5% of the adult population in 2010, which increased by 155% in the period from 2010 to 2016 (Centre for Diabetes and Endocrinology 2016; Ramkisson, Pillay & Sartorius 2016).

As persons living with DM2 are at risk of all parts of the body being potentially affected, those who do not take care in regulating their blood sugar levels have a significant chance of developing other conditions and complications. These include nephropathy, leading to renal failure; retinopathy, leading to blindness; and circulatory problems, leading to lower limb amputations, neuropathy and coronary artery disease (Pheiffer et al. 2018).

The range of comorbidities that may accompany DM2 has major implications for healthcare budgets in both developed and developing countries. In most African countries, the healthcare services are burdened by a range of communicable and non-communicable diseases, the management of which drains the available public healthcare resources (Hofman 2014; Mendenhall & Norris 2015). It is reported that the IDF estimates that South Africa has a ‘mean health expenditure per person with diabetes of USD 918.9’ (R12825.65) per annum (Society for Endocrinology, Metabolism and Diabetes of South Africa [SEMDSA] 2017:S9). Poverty and lack of information resources, together with the low socio-economic environment which some persons living with DM2 find themselves in, lead to lowering of morale and encouragement of ‘negative lifestyle choices’, such as a sedentary lifestyle with poor nutrition choices (Houle et al. 2016).

South Africa has become known as an obese nation, which is a major contributory factor in the increase in the incidence of DM2 (Adam & Rheeder 2017; Hofman 2014). Obesity may lead to an increased insulin resistance, predisposing the individual to the development of DM2 (Unnikrishnan et al. 2017). Communicable diseases, such as human immunodeficiency virus (HIV) and tuberculosis (TB), may also be contributory factors to this increase as there are often negative drug interactions between medications used for the management of DM2 and TB (Sahadew, Singaram & Brown 2016) and those used for the management of DM2 and HIV (Mendenhall & Norris 2015).

Some published studies have usually concentrated on giving an in-depth insight into the patient experience of living with DM2 (Johansson et al. 2018; O’Brien, Van Rooyen & Ricks 2015; Pikkemaat, Boström & Strandberg 2019), but there is little evidence available on the experiences of diabetes nurse educators who provide patient education and support, particularly with regard to coordination of care and lifestyle modification. However, the positive role of diabetes nurse educators in this regard is without question (Ginsberg, Hoffman & Azuri 2017; Johansson et al. 2018; Riordan et al. 2017).

This study formed part of a larger research study undertaken in Nelson Mandela Bay Municipality aimed at the development of strategies that could be used by diabetes nurse educators in facilitating self-management of DM2 of persons living with DM2, and it was also described in a previous article (O’Brien et al. 2015). The first author undertook the study under the supervision of the second and third authors. The purpose of this article is to describe the experiences of diabetes nurse educators in relation to promotion of self-management by persons living with DM2.

## Research methods and design

### Study design

A qualitative, descriptive, exploratory and contextual approach was used. For this part of the larger study, the experiences of diabetes nurse educators in facilitating self-management of DM2 of persons living with DM2 were explored and described.

### Setting

The specific context for this study is Nelson Mandela Bay, South Africa. Nelson Mandela Bay is one of the eight metropolitan municipalities in South Africa, consisting of the towns of Port Elizabeth, Uitenhage and Despatch; it lies in the western region of the Eastern Cape province. It is an industrial area where manufacturing is an important source of formal employment (Eastern Cape Department of Economic Development, Environmental Affairs and Toerism 2017, Annual Report 2017 and 2018) https://provincialgovernment.co.za/department_annual/736/2018-eastern-cape-economic-development-environmental-affairs-and-tourism-annual-report.pdf Date of access: 2 November 2020

It was estimated in 2016–2017 that there was an incidence of DM2 in 1.7 per 1000 population in Nelson Mandela Bay, which has a population of 1.152 million (Health Systems Trust 2018, Annual Health Review 2018, Health Systems Trust, Johannesburg). However, at the time of this study, little research had been conducted on DM2 in the area. This study was undertaken in an attempt to provide an insight into aspects of assisting those living with DM2 in Nelson Mandela Bay.

### Study population and sampling strategy

The study population consisted of professional nurses working as diabetes nurse educators in Nelson Mandela Bay. Diabetes nurse educators work at diabetes clinics at each state hospital, as well as with private practice medical doctors with a special interest in diabetes. A purposive sampling was used as there are a limited number of diabetes nurse educators in the Nelson Mandela Bay metropolitan area who met all the required criteria. Participants had to:

1. be formally registered with the South African Nursing Council (SANC) as a professional nurse
2. have a minimum of 1 year experience as a diabetes nurse educator in either private or public healthcare facilities
3. have additional training or courses completed in diabetes education
4. have worked in either the public or the private sector.

Participants were recruited via a key informant – a diabetes nurse educator working with a local physician. A list of potential participants was given to the first author who contacted each potential participant telephonically.

### Data collection

A focus group session with three participants was held at the researcher’s place of employment, facilitated by the researcher. A date convenient to most participants was communicated both telephonically and in writing. Unfortunately, four potential participants were unable to take part on the day because of unexpected work commitments and two withdrew. However, individual interviews were conducted with two of the four potential participants who could not participate in the focus group interview at a later stage. Thus, a total of five diabetes nurse educators participated in the study. The researchers and independent coder were satisfied that data saturation was achieved. The research objectives and process were explained to the participants, along with ethical considerations. Participants were given the opportunity to withdraw if they wished to do so. Each participant completed an informed consent form and was asked to give consent for the session to be recorded.

A semi-structured interview guide was used by the researcher for the focus group session, with the following questions:

1. What do you perceive as effective self-management in patients with DM2?
2. What factors do you think assist patients in managing their condition?
3. What factors do you think hinder patients in the management of their condition?
4. How can professional nurses assist diabetic patients in enhancing self-management of DM2?

The session was facilitated by the first author using a voice recorder. The participants demonstrated their passion for their work and their patients by their enthusiastic discussion of the research questions. Communication during the interviews was both verbal and non-verbal as the first author observed body language and facial expressions that remained positive. Questions were based on the semi-structured interview guide with some probing questions, as well as clarification and reflection when required. Field notes were recorded by the first author after each interview.

Over the next week, two individual interview sessions were held with diabetes nurse educators who were unable to attend the focus group session but expressed a desire to take part in the research project. The same procedure was followed regarding explanation of the research process and completion of the informed consent form. The same semi-structured interview guide was used and the interviews were recorded with their permission.

All participants responded well to the questions asked during the interviews. As the responses provided by participants in both the focus group interview and the individual interviews began to be repeated with no new material forthcoming, and as the data collected were similar to that collected from persons living with DM2 during the larger study mentioned previously, it was deemed that data saturation had been achieved. This was decided in a discussion with the second and third authors, both experienced in qualitative research.

### Data analysis

The tape recordings were transcribed verbatim by an independent transcription typist, providing a complete record of the conversations from the focus group and individual interviews.

Coding commenced immediately upon completion of transcription by using Tesch’s eight-step method (Cresswell 2003). The steps identified by Tesch (1990) as the most useful method of providing data analysis begin by reading and rereading the transcripts to get a sense of the whole, making brief notes on topics that come to mind during this step. Similar topics were then clustered and codes were assigned to them. The coded topics were grouped into categories to help identify any inter-relationships. Data were assembled for each category and a final decision was made on the themes and sub-themes. The steps taken in this study are described in Table 1. Using the themes identified, it was possible to create a picture of the experiences of the diabetes nurse educators who participated in this study.

**TABLE 1:** Tesch’s method of data analysis.

Data analysis – Tesch’s method	Steps taken in this study
Read all transcriptions carefully to get a sense of the whole	It is important to ensure that all transcriptions are read before undertaking the detailed analysis.
Choose one document for further analysis of underlying meanings	The focus group interview (FG) was chosen for commencing the detailed process.
Continue this process for several documents and then make a list of topics, clustering those which are similar	The lengthy list of initial topics was gradually reduced by clustering similar topics.
Assign codes to the topics	Return to the data and assign codes to the appropriate segments.
Assign categories to the topics, grouping them and indicating relationships	As the topics were categorised, a number of themes emerged.
Decide on an abbreviation for each category	For example, DNE/Assist or DNE/Hinder for those factors that participants felt either assisted or hindered the self-management of the condition.
Perform a preliminary analysis by assembling the data for each category together	Assembling data for each category allowed the themes and sub-themes to emerge.
If necessary, recode existing data	Further reading and analysis of the data resulted in the themes as described in this article.

*Source*: Cresswell, J.W., 2003, *Research design: Qualitative, quantitative and mixed methods approaches*, 2nd edn., Sage, Thousand Oaks, CA.

### Trustworthiness

Trustworthiness in qualitative research is established by a variety of constructs, namely, credibility, transferability, confirmability and dependability, which are used in accurately representing the experiences of the participants. Credibility refers to an accurate description of the findings for it to be judged and believed to be credible. Member checking was carried out and a semi-structured interview schedule ensured that all participants were asked the same questions. Transferability was ensured by providing a detailed description of the particular context of the study because it enhances the level of transferability. Coding was completed by the first author and an independent coder who assisted in ensuring the confirmability of the research findings. Dependability was ensured by confirmation of all themes and sub-themes identified during the data analysis process through discussion with the second and third authors, as well as the independent coder. The analysis and interpretation of findings were supported by documentation from at least two sources in the report of the research findings.

### Ethical considerations

Ethical guidelines laid down by Nelson Mandela Metropolitan University (now Nelson Mandela University) were adhered to during this study. Prior to commencing this study, approval was obtained from the Research Ethics Committee of Nelson Mandela University (clearance number: H07HEANUR-001).

Ethical considerations addressed in the study included the need to ensure that there was no harm or deception to participants and no violation of privacy, the need to obtain informed consent and the necessity to maintain confidentiality. These principles were upheld as follows: to prevent any harm to participants, both physical and emotional, the procedure and implications of the study were explained to each participant before the interviews took place. Written informed consent was obtained with specific consent to audio record both the focus group session and the individual interviews. The participants were able to ask for further information if they wished to do so. A code, known only to the researcher, was allotted to the data from each participant. The resulting data could not be linked to the individual in any way. Interview tapes were stored in a locked safe apart from the transcripts and would be destroyed after 5 years as per the institutional policy.

## Results

Participants included in this study were mainly from the private healthcare sector. One participant worked in both sectors at the time the interviews were conducted. However, all participants had experience of working in the public sector. Two participants worked mainly in lower socio-economic areas and, although presently employed in the private sector, had worked in a similar role for many years in the public sector. Their length of experience as diabetes nurse educators varied from 19 months to 16 years. As a group, the participants had contact with a wide range of patients from a variety of socio-economic groups. All participants were women.

The following themes were identified during data analysis:

- Diabetes nurse educators had clear perceptions about the importance of self-management of DM2 in persons living with DM2 in terms of patient education, motivation and empowerment of persons living with DM2.
- Diabetes nurse educators had clear views on factors that affect persons living with DM2 in self-management.
- Diabetes nurse educators identified ways in which professional nurses can assist persons living with DM2 in the self-management of their condition.

The major themes identified in the data provided by the diabetes nurse educators are described next.

### Theme 1: Diabetes nurse educators had clear perceptions about the importance of self-management of diabetes mellitus type 2 of persons living with diabetes mellitus type 2 in terms of patient education, motivation and empowerment of persons living with diabetes mellitus type 2

The experienced diabetes nurse educators who participated in the interviews had clear perceptions about the importance of self-management of DM2 in persons living with DM2, as well as of the role professional nurses (who may not necessarily be trained as diabetes nurse educators) can play.

Patient education was recognised as the basis of diabetes nurse educators’ work as it provided persons living with DM2 the knowledge required to better manage their condition. With increased knowledge, the person living with DM2 is able to make informed choices regarding the various aspects of self-management of the condition. This was confirmed as:

‘Knowledge is really power and knowledge makes management so much better.’ (FG)

‘… [*E*]very patient should be taught right from the word when they’re diagnosed with diabetes to take responsibility for themselves … They must learn about diabetes.’ (Diabetes Nurse Educator 1)

‘There’s certain things that the patients need to know and need, people they need to see, to put them onto this road of self-management and having a goal and being motivated. There’s nothing as de-motivational I think, as not knowing why you need stuff.’ (FG)

Lifestyle choices have to be made by each individual to improve and maintain an optimal level of self-management. The choices involve activities that include self-monitoring of blood glucose, dietary control, adjustment of insulin dose to actual needs, correct administration of oral medication, weight control and regular exercise. Once again, patient education assists in motivating and empowering persons living with DM2 by enabling them to make better choices for their own condition and lifestyle:

‘It is a patient that is well-educated; he is well-motivated; he’s got his goals set and he is trying to reach them.’ (FG)

‘The main aim in managing a diabetic is to keep them as long as possible complication-free.’ (DNE 2)

All the participants agreed that diabetes nurse educators are in a position to positively affect the level of self-management achieved by the person living with DM2, whilst preventing burnout resulting from the burden of living with the condition. Some persons living with DM2 find living with the condition a huge burden and may not believe that they are able to achieve an optimal level of self-management in spite of perceived personal barriers. The participants emphasised the importance of their role in breaking down the barriers to optimal self-management:

‘It is a big struggle for those other type of patients that I’m getting, because you need to convince them.’ (FG)

‘The more we make people aware of how to treat diabetes, the more patients will be able to empower themselves.’ (FG)

As part of the education process, participants indicated that they need to encourage those they deal with to take responsibility for their own management. This empowers patients to take control over their own lives:

‘… [*R*]ight from diagnosis, they must look after themselves, they must learn about diabetes, they must know what the complications are ….’ (DNE 2)

‘… [*I*]f they know everything about their disease and what can happen, then they will most, or easier, take responsibility for their condition.’ (DNE 1)

From the above quotations, it is evident that knowledge is recognised by the participants as the underlying factor that assists persons living with DM2 to come to terms with their condition and attain optimal self-management. The level of knowledge of the individual on all aspects of the condition will affect their ability to take responsibility for their own self-management.

In view of the above research results, it is evident that the first major theme in this study emphasises the importance of patient education, motivation and empowerment in gaining and maintaining a high level of self-management in persons living with DM2.

### Theme 2: Diabetes nurse educators had clear views on factors that affect persons living with diabetes mellitus type 2 in self-management of their condition

The second theme identified was that the diabetes nurse educators participating in this study all experienced factors in their interactions with persons living with DM2, which may affect those persons in, either negatively or positively, achieving optimal levels of self-management. Some of the factors had positive effects, but most seemed to have a negative effect on the self-management level achieved by persons living with DM2.

Accepting responsibility for their own condition and management is difficult for some people diagnosed with DM2. Denial is a normal part of the initial reaction to the diagnosis, but denial normally begins to fade as acceptance of the condition is achieved (Silva et al. 2018). However, the participants experienced that some persons living with DM2 remain in denial, making self-management a difficult goal for them to achieve:

‘On an emotional level, they’re not taking it, they’re in denial, they don’t take it right, and so they can refuse it with certain things.’ (FG)

‘It’s no good if they’re in denial, you’re not going to get anywhere, they must accept that they’re diabetic.’ (DNE 1)

‘The minute they realise that they’re in control of their condition, then things go better. So, they must have an open mindset first and then things fall into place.’ (DNE 1)

Physical problems as well as other medical conditions may affect the ability of the individual to achieve an optimal level of self-management. Examples mentioned during the discussions included bad eyesight and the perceived inability of starting and maintaining an exercise programme:

‘They can’t see most of them because they’ve got eye problems so they don’t know how much to draw up ….’ (DNE 2)

‘I always tell them exactly that gym is not the only answer because in fact how many people can afford to go to gyms.’ (DNE 2)

‘I always tell the patients that even if you sit in your wheelchair and you haven’t got legs and you sit and you move your arms, really everything that makes your pulse rate go up is a form of exercise.’ (DNE 1)

In South Africa, we have many cultural groups in our communities who often differ on all aspects of life, including healthcare. Social influences may play either a negative or a positive role in influencing the level of self-management attained by the person living with DM2. Participants in this study deal with patients from many cultural groups and so have first-hand experience of this. Some cultural groups regard diabetes as a curse that may cause the person living with DM2 to remain in denial about the condition, whilst others may prefer to go to traditional healers, for example, sangomas rather than medical doctors trained in developed countries:

‘In the Indian community, the main thing they’re saying: It’s a curse.’ (DNE 2)

‘They do go to sangomas, my dear. You are the last door that they knock at.’ (FG)

During the focus group session, there was some discussion between participants about certain cultural traditions:

‘There was a stage when Xhosa males, especially don’t want insulin … It’s a sign of weakness if you have to inject. Is it a thing that’s still alive?’ (FG)

‘… [*Y*]ou still find it in most of the patients … they believe in taking the tablets but when it comes to insulin, it’s not so easily acceptable. … “People that use insulin die” … That’s what they believe in.’ (FG)

Cultural aspects can also play a role in the ability of diabetes nurse educators to provide support in other areas, such as that of sexual dysfunction occurring as a complication of DM2:

‘With the barrier of being a female talking to a Xhosa male, I can’t go directly there.’ (FG)

However, it is important that the matter is addressed as it can have serious negative consequences for relationships, leading to suspicions of infidelity from the spouse of the person living with DM2. All the participants reported this as an experience that has to be dealt with as part of the counselling process.

Participants expressed their concern that some patients have a lack of financial and other resources that may make self-management difficult. Lack of access to resources may be owing to a variety of reasons, such as transport difficulties, unawareness of the availability of a particular resource and limitations imposed by medical aid schemes. An example given was the issue of transport that can be expensive particularly for those living with limited financial resources. A participant highlighted the transport problem for some persons living with DM2 as follows:

‘They sometimes have to take two taxis and a bus to get there.’ (DNE2)

However, another participant pointed out that some individuals prefer to visit the hospital instead of a clinic that is closer to them in spite of transport costs:

‘Actually they could have gone to a nearer place where it’s in walking distance and could have got their medication. You try and explain it to them, then they tell you I’m a patient of this hospital because I prefer to come here.’ (DNE1)

Support groups are not always available to the individual – the patient may not be aware of available groups or may not be able to attend owing to, once again, transport problems. Because of the breakdown in the traditional family structures, which has taken place in the South African society, not all persons living with DM2 have the family support that they may have enjoyed in the past. All participants in the focus group expressed the importance of support for optimal self-management, as well as concern about the lack of support. They were particularly concerned about the lack of family support experienced by some persons living with DM2, as expressed in the following quotes:

‘Lack of support. Lack of family support ….’ (FG)

‘You need to have something to encourage the patient with … if the partner is there, we speak to both of them.’ (FG)

One of the resources discussed by study participants was medical aid (medical insurance). To achieve an optimal level of self-management, persons living with DM2 require access to medication, testing strips and lancets for checking their glucose levels, and to regular checks for damage caused by the condition. Checks by ophthalmologists, podiatrists, dietitians and physicians should be performed at least on an annual basis. Access to medical aid and treatment in the private sector was not a guarantee of being better off in terms of self-management of DM2 as, according to participants’ reports, the coverage provided by some medical aid schemes was not always sufficient for the needs of the particular patient.

A participant compared the service offered by her place of employment with that of another service that offered a high level of service, but was only accessible to members of a limited number of medical aids:

‘You see because the service is only available to the people that are on certain medical aids, … but in our practice, we treat our diabetics in exactly the same manner as if they are on. … The only other thing is they then have to pay out of their funds or privately when we send them to the podiatrist or the dietitian.’ (DNE 2)

Discrepancies in levels of supplies and service were a constant source of frustration for the participants. They also resented the fact that medical aid schemes often dictate the treatment to be offered to the patients in their care:

‘Medical aids these days prescribe to the doctors and I don’t know how they work it; but if a doctor decides which insulin would be better for the patient, the medical aid will disagree.’ (DNE 2)

The limitations imposed by some medical aid schemes and by the public health services apply, in particular, to the supply of insulin in vials to be used with syringes and needles instead of the more easily used insulin pens:

‘It’s not cost-effective … because they need a syringe plus the vial – Whereas, if they’ve got the pen … and there’s not that much difference in price.’ (DNE 2)

Medical aids are required by law to provide treatment for a number of chronic conditions, known as prescribed minimum benefits (PMBs). One such condition is DM2; therefore, legislation regarding PMBs assists patients living with DM2 in this matter, but medical aid schemes are not required to supply the latest medication or treatment for PMBs (Council for Medical Schemes 2010, 2016). Some medical aids, however, play a positive role with programmes for chronic conditions:

‘What actually also is a big help for a patient managing is because of the legislation about prescribed medical benefits for certain 25 diseases and diabetes obviously being one of them, at least you’ve got some foot to stand on, … A lot of my time is going in dealing with medical aids.’ (DNE 2)

‘There are medical aids who actually force their patients to come. They are forced to go on [*the programme*] because they’re saving a lot of money.’ (DNE 2)

Access to information sources is another barrier to effective self-management of DM2, which was identified by study participants. The participants experienced difficulty in obtaining adequate, comprehensive and culturally sensitive printed brochures or booklets for the persons living with DM2 they encounter in the course of their work:

‘Because in the libraries and the bit of information that’s there, is so outdated and your um, internet’s very good but most of the products that are there on the internet is not available in South Africa.’ (DNE 2)

‘We have very little South African information that comes out. We don’t have enough South African information that is available to the patients.’ (DNE 1)

For a holistic approach to the problem of self-management, the person living with DM2 should be able to have access to specialist care when required. However, some participants felt that it appears that some general practitioners find it difficult to refer their patients to a specialist or to other members of multi-disciplinary teams. This reluctance to refer patients affects self-management as it deprives the person living with DM2 of a wider range of resources and support that could assist in achieving better glycaemic control:

‘[*General Practitioners*] in general … At the present moment they’re not very keen on referring and the minute they do refer is when the patient’s got complications already.’ (DNE 1)

‘They don’t make use of the other team-members – like dieticians and educators and things like that, they just carry on’.’ (DNE 1)

In both the focus group session and the individual interviews, the participants showed their concern for those persons living with DM2 they encountered on a daily basis.

The descriptions of the various factors that were identified during both the focus group session and the individual interviews gave a clear picture of what can facilitate or hinder persons living with DM2 in the process of achieving optimal levels of self-management. The factors identified included psychological and physical problems, cultural issues, and financial and medical aid issues, as well as a lack of relevant information.

### Theme 3: Diabetes nurse educators identified ways in which professional nurses can assist persons living with diabetes mellitus type 2 in the self-management of their condition

Diabetes nurse educators made several suggestions to address the shortfalls in the service that is provided by professional nurses who are not trained diabetes nurse educators to improve the assistance they provide to patients regarding the self-management of DM2. Professional nurses encounter persons living with DM2 on a daily basis in the course of their normal work.

Participants felt very strongly that all professional nurses need to keep up to date on the new developments in the management of DM2. Research takes place on a continual basis, which will lead to new methods in dealing with issues that may arise. Pharmacology was identified as being of particular importance:

‘The nurses must educate themselves first, before they can educate patients … Because if you don’t have the knowledge you can actually do more harm to the patient than to help them.’ (DNE 2)

‘Because 95% or 98% of the staff, trained staff that’s working with diabetes, hasn’t got the vaguest idea of what’s really going on. How to know the action of the medication and things like that … They just hand it out.’ (DNE 1)

‘I had a guy the other day that came to me, he’s on insulin. He’s been on it for 6 months, so I said “Right, where do you give it?” “Oh, no, I spray it in my mouth.” He didn’t know! He was always taking his tablets, nobody said to him. “This is now an injection, you’ve got to inject yourself”.’ (DNE 2)

Diabetes nurse educators as well as professional nurses must bear in mind the financial status of the person living with DM2 as the socio-economic status of the individual affects their ability to maintain the required standards of self-management. Financial problems may be a sensitive issue that needs to be addressed during interactions between the diabetes nurse educator and the person living with DM2. In order to maintain adequate glycaemic control, persons living with DM2 are confronted with potentially expensive medication regimes and may find that they must spend a higher proportion of the family budget on healthier food options, if they previously followed an unhealthy diet. In the private sector, medical aids affect the level of care available to an individual. Unfortunately, there are many people who are unable to afford the medical aid payments and have to make use of public healthcare services. Transport difficulties and being unaware of the availability of resources also affect the level of care available:

‘The service is only available to the people that are on certain medical aids.’ (DNE 2)

‘Most of our patients unfortunately today are falling into the public sector, we can’t afford medical aid for ourselves even you know.’ (FG)

An example of services that may be required by the patient is psychological support as depression is a common occurrence, particularly when the person does not accept the diagnosis. The professional nurse must be able to recognise the need for psychological support and to act as quickly as possible. Furthermore, the professional nurse must be aware of services that could be accessed by the person living with DM2 and ensure that they are referred to those services that ensure holistic care when required:

‘We immediately ask doctors to refer them to a psychologist because unless they’ve accepted the fact that they’re diabetic, they won’t get compliance.’ (DNE 1)

‘A dietitian … a podiatrist once a year, an eye specialist once a year, all the necessary things’ [*when required*]. (DNE 1)

The participants were adamant that diabetes should receive the same recognition as other chronic conditions, such as HIV and acquired immunodeficiency syndrome (AIDS). Diabetes mellitus type 2 is a major and growing epidemic worldwide and has a strongly genetic element. However, participants felt that this is forgotten and that the emphasis is given to conditions such as HIV and AIDS:

‘How many of our diabetics, what have they done wrong to become a diabetic? If you look at the world stats, I’m sure you agree, it’s just the same. But, I mean, how much money gets really spent on diabetics? It just gets treated as a by-the-way thing, and not the big fuss they make about HIV.’ (DNE 1)

‘… [*T*]hey [*Government*] pay for AIDS counsellors by the hundreds, by the thousands of rand – why don’t they pay for diabetic counsellors? If you look at our people in government … They’re not doing anything about diabetes – nothing!.’ (DNE 2)

In the above presentation of research findings, the participants indicated the importance of the role of professional nurses in assisting persons living with DM2. They also indicated their concern about the need for extra training of professional nurses in order that they may remain up to date regarding new research and methods to be used in the management of the condition with a particular emphasis on pharmacology training related to the drugs used in the treatment of DM2. The participants shared that the professional nurses providing services to DM2 patients should be sensitive to financial aspects of their patients and should also be aware of all services required by the person living with DM2.

## Discussion

The participants in this study drew from a wide range of experiences in describing their perceptions of optimal self-management of DM2 of persons living with DM2 and the factors that facilitate or hinder the attainment of optimal self-management. They also drew from their experience in identifying areas where professional nurses could assist persons living with DM2 with self-management issues. Analysis of the themes described above provided an insight into the experiences of diabetes nurse educators whilst assisting persons living with DM2 with issues regarding self-management.

The first theme showed the importance of patient education particularly with regard to motivating and empowering persons living with DM2. Whilst interacting with patients, the participants indicated that they could assist with the self-management through patient education, which can in turn motivate and empower persons living with DM2.

The importance of this role has been confirmed in several studies that have shown the value of the educational role of a registered nurse/case manager in relation to diabetes and other chronic conditions (Forjuoh et al. 2014; Ginsberg et al. 2017; Riordan et al. 2017).

The basis of patient empowerment for persons living with DM2 is an understanding of the inter-relationships between the activities of self-management and the ability to implement appropriate changes when required. Studies have shown that efforts to empower persons living with DM2 have many positive effects, including health benefits for the individual and major cost savings for all concerned (Ginsberg et al. 2017; Johansson et al. 2018).

To prevent an information overload that is counterproductive, the person living with DM2 should be given the opportunity to take part in an educational process that considers the needs of the individual. Patient education can take place on an individual level, which is the usual manner for the participants, or on a group basis, but should be directed at setting goals that will assist behaviour change and encourage the patient to adhere to the principles of self-management as described in several studies (Ginsberg et al. 2017; Johansson et al. 2018; Riordan et al. 2017).

Persons living with DM2 require motivation to implement the required lifestyle changes, whether they are newly diagnosed or have been diabetic for many years (Ramkisson et al. 2016). By developing a therapeutic relationship with persons living with DM2 and providing them with the required information and guidelines they need, the diabetes nurse educator empowers them to play an active and informed role in the decision-making process regarding their own care (Mendenhall & Norris 2015). However, the participants felt that the diabetes nurse educator has to realise that not all persons living with DM2 are able to or want to take control of their own management and the education process has to be adjusted accordingly. The need for individualised patient education has been highlighted in the literature (Ramkisson et al. 2016), particularly as a means to prevent or manage diabetes-related distress.

Literature reflects that a lack of knowledge is often found to be one of the most frequently reported barriers to achieve optimal self-management in DM2 (Riordan et al. 2017), which was confirmed by the participants. Studies have shown that goals set during education sessions motivated the recipients to adjust their behaviours and improve adherence to a self-management programme, as well as to improve empowerment in the individual (Ginsberg et al. 2017; Johansson et al. 2018).

A person living with DM2 who has undergone an appropriate education programme and has developed a high level of personal responsibility should be empowered enough to request a referral to an appropriate multi-disciplinary team member if required. All participants agreed that in their experience, people, including those living with DM2, are questioning their medical practitioners more often regarding the proposed treatment as they become more knowledgeable, particularly if they make use of resources, such as the Internet, to find out more about their conditions. The literature shows that the use of the Internet for healthcare information is increasing in popularity, although the usage rate varies according to age and socio-economic status (Wong et al. 2014). Internet sources can be a useful aid for health promotion and patient education, but can be misleading or may lead to misinterpretation if the individual carrying out the search has a low level of health literacy and is unable to assess the trustworthiness of a website. This is an important issue that should be taken into account when commencing an education programme on self-management of DM2.

The second theme that was identified highlighted factors that may either assist or act as a barrier to achieving a high level of self-management by the person living with DM2. In an education programme that addresses the needs of the individual, the professional nurse has the potential to perform an important motivational role by identifying and addressing barriers to self-management of DM2 that are perceived as daunting, a factor that the participants agreed they experience often in their practice. This is reflected in the literature where even in countries regarded as developed nations, similar barriers to self-management of DM2 have been experienced (Mendenhall & Norris 2015; Riordan et al. 2017).

Barriers identified by the diabetes nurse educators included access to healthcare services, financial factors, lack of personal responsibility on the part of the patient and denial about the condition, physical factors including possible comorbid conditions such as blindness, retinopathy, nephropathy, angina, stroke and microvascular conditions leading to amputation as described in several publications (Houle et al. 2016; Ramkisson et al. 2016). Cultural concerns may also play a significant role in creating barriers, particularly in communities where there is a stigma attached to the diagnosis as pointed out by the participants. Another barrier that participants identified is the observation that some doctors appeared to be reluctant to refer patients for specialist care when required. This is not a new phenomenon – a study undertaken in Norway (Thorson et al. 2016) showed a similar reluctance by some general practitioners to refer patients to specialist services.

The provision of adequate health services for all citizens is a challenge for governments globally. Even where health services are provided in a ‘free at the point of delivery’ model, such as the public health sector in South Africa, access to those services may be limited by factors such as accessibility and availability (Burger & Christian 2018). Participants experienced that although the service may be free, factors such as transport costs, staff shortages and lack of equipment or medication at the service provider can make the services inaccessible for the most vulnerable groups (Ramkisson et al. 2016). On the other hand, a private healthcare model is expensive, and many people are excluded simply because they do not have enough money which the participants felt was unfair. The proposed National Health Insurance scheme for South Africa is an attempt to address these issues and to make higher quality health services more accessible to all (South Africa 2018).

If a person living with DM2 is unable to access healthcare services, for whatever reason, resources may be said to be wasted. If the potential user is unaware of the availability of resources, once again, they may be said to be wasted (Mendenhall & Norris 2015). The participants in this study believed that accessing healthcare resources may be a significant problem for many people in the Nelson Mandela Bay area, thus affecting the level of self-management achieved by those persons living with DM2. A study describing difficulties and barriers in providing patient-centred care in public hospitals in Nelson Mandela Bay (Jardien-Baboo et al. 2016) confirmed this belief.

Financial implications of DM2, in terms of mentioned medication and lifestyle modifications, can become burdensome for the individual, leading to higher levels of diabetes-related distress. The level of healthcare provided to the individual depends on the rules of the medical aid scheme or the public healthcare facility utilised. Studies in South Africa and internationally have shown that socio-economic factors play a significant role in the level of care received by the individual (Landau & May 2013; Ramkisson et al. 2016). As a result, diabetes nurse educators need to know where to refer persons living with DM2 who require extra assistance. Participants felt that dealing with medical aids is a major part of their jobs. The situation is changing, however, as medical aid schemes are making use of chronic condition programmes for their members. These programmes provide the person living with DM2 with a coordinated monitoring programme that assists in self-management issues. They also provide guidelines that both doctors and the diabetes nurse educator may use in assisting the individual person living with DM2 (SEMDSA 2017).

Adequate patient education on all aspects of DM2, as well as the risk of complications, does not guarantee compliance and adherence to the required lifestyle changes, which is frustrating for the participants. However, they acknowledge that the diabetes nurse educator must recognise that some people are unable to or refuse to acknowledge their diagnosis and do not wish to take responsibility for self-management of their condition, which has been confirmed by studies undertaken overseas (Ginsberg et al. 2017; Johansson et al. 2018).

As the diabetes nurse educator is expected to fulfil the role of a counsellor, an empathetic attitude is essential, and each patient should be treated as an individual. In the experience of the participants, this can be difficult when a lack of time, combined with a large patient load, makes effective communication between nurses and their patients challenging.

Cardiovascular disease, renal disease, blindness and lower limb amputations are examples of comorbidities and complications that may affect patients’ quality of life. Management of these conditions forms an important part of the assistance provided by the diabetes nurse educators participating in this study. Literature reflects that comorbid conditions may have a serious detrimental effect on the quality of life experienced by the person living with DM2, as well as on any attempts to maintain an optimal self-management programme (Hofman 2014).

As South Africa is a multi-cultural society, participants agreed that it is essential that special attention is given to cultural factors that may affect the implementation of lifestyle changes, especially about diet, in the person living with DM2. A number of cultural factors affecting self-management of DM2 have been described in the literature, including the stigma associated with the condition that may be found in some communities (Houle et al. 2016; Mendenhall & Norris 2015). In certain communities, having an overweight wife is a sign of prosperity, which, in the experience of the participants, makes the promotion of healthy eating habits very difficult. People who lose weight may be stigmatised as community members may conclude that they have been infected with HIV and/or AIDS. Furthermore, in a multi-cultural society, cultural attitudes affecting access to and utilisation of healthcare services – especially regarding diabetes care – need to be understood by anyone providing health services. The importance of showing respect in dealing with various cultural groups has been described in the literature and is not only relevant to South Africa (Caliari et al. 2017; Ginsberg et al. 2017).

Particularly in the public sector healthcare services, the provision of adequate and up-to-date equipment can be very erratic. The study participants who had worked in the public sector mentioned that this is an ongoing problem. Although some facilities have state-of-the-art equipment, others may be in short supply of basic equipment, such as sphygmomanometers (O’Brien, Van Rooyen & Carlson 2006). Glucometers, scales, sphygmomanometers and diagnostic sets, which should be readily available for correct patient assessment, may be missing or not working, providing a constant source of frustration for diabetes nurse educators working in such facilities. This was confirmed by the participants who had all previously worked in the public sector and had first-hand experience of the lack of adequate equipment.

The third theme that was identified deals with the importance of professional education and training. The participants pointed out that it is the responsibility of each professional nurse, including diabetes nurse educators, to ensure that they continue to educate themselves about new developments and technology. Diabetes mellitus type 2 is a highly complex condition that is continuously being researched, resulting in the publication of information that may change the way of doing things. If diabetes nurse educators are encouraged to identify their own shortcomings in skills and knowledge, relevant educational programmes should be made available to improve patient care and reduce expenditure on healthcare (Johansson et al. 2018).

All participants of this study acknowledged the need for extra training on the pharmacological aspects of self-management. They pointed out that particular attention should be given to interactions of medications, especially when the person living with DM2 suffers from more than one chronic condition. As each class of drugs acts on the body in a different manner, the effects, potential complications and side effects will also differ (Riordan et al. 2017).

No accredited courses on the management of diabetes are recognised by the SANC (De Medonca 2009). Short intensive courses are available, such as that offered by the Centre for Diabetes and Endocrinology in Johannesburg, which two participants had completed (Centre for Diabetes and Endocrinology 2019). This may change as healthcare services are confronted with a double burden as patients present with HIV and AIDS together with other chronic non-communicable diseases such as DM2 and related conditions, including cardiovascular disease (Pheiffer et al. 2018). Participants emphasised the importance of further training that will be required to deal with this burden, preferably at a tertiary level, at institutions approved by the SANC. They also strongly felt that post-basic courses should include aspects of DM2 where relevant.

## Strengths and limitations

The findings of this study provided valuable insights into the experiences of diabetes nurse educators regarding how persons living with DM2 self-managed their condition and were vital in developing the action, co-ordination and education (ACE) strategies aimed at facilitating diabetes nurse educators in their role of assisting persons living with DM2 in the self-management of the condition. The ACE strategies were described in a previously published article (O’Brien, Van Rooyen & Ricks 2018).

A limitation of this study was the lack of input from nurses without specialised training in the management of DM2. All participants in both the focus group session and the individual interviews were experienced diabetes nurse educators. Another limitation was the lack of input from nurses presently working in the public sector. However, all participants in this study had previously worked in the public sector and one is currently employed at a local public hospital. Professional nurses currently employed in the public sector were invited to take part, but were unable to do so.

There was no input from professional nurses working in other specialities who do not have specialised training in assisting persons living with DM2. As all professional nurses do encounter persons living with DM2 in their work, their input would be useful in identifying the educational needs of those who do not have specialised training.

## Recommendations

The following recommendations were made by the participants of this study:

Specialised training courses, preferably at tertiary level, should include methods of facilitating the development of personal responsibility and empowerment of the person living with DM2, reinforcing positive memes regarding health behaviour. These courses should be made more readily available to those working as diabetes nurse educators. Regular in-service education and training on all aspects of self-management of the condition should be available so that staff working with persons living with DM2 can be kept updated with new developments and research.

Similar studies targeting other professional groups in the multi-disciplinary team – such as dietitians, podiatrists, medical doctors, ophthalmologists and physiotherapists – may provide insights that will enhance the collaborative effort in dealing with the self-management of DM2.

## Conclusion

In describing their experiences, the diabetes nurse educators participating in this study were able to create a picture of the realities faced whilst assisting persons living with DM2 in the self-management of their condition. The participants identified factors that both assist and hinder patients in self-management and also voiced their views on how professional nurses can support these patients. The experiences described by the diabetes nurse educators are not unique to South Africa, as shown by both national and international research. This article provided various recommendations that can assist both diabetes nurse educators and nurses working with patients with DM2 in meeting these challenges.
